# Successful Reconstruction of the Right Ventricular Outflow Tract by Implantation of Thymus Stem Cell Engineered Graft in Growing Swine

**DOI:** 10.1016/j.jacbts.2019.02.001

**Published:** 2019-06-24

**Authors:** Ambra Albertario, Megan M. Swim, Eltayeb Mohamed Ahmed, Dominga Iacobazzi, Michael Yeong, Paolo Madeddu, Mohamed T. Ghorbel, Massimo Caputo

**Affiliations:** University of Bristol, Bristol Heart Institute, Bristol, United Kingdom

**Keywords:** congenital heart disease, reconstruction, right ventricular outflow swine model, tissue engineering, tract stem cells, CM, cardiomyocyte, cMYH, cardiac myosin heavy chain, Cx-43, connexin-43, DMEM, Dulbecco’s modified Eagle’s medium, EC, endothelial cell, FBS, fetal bovine serum, IL, interleukin, IsoB4, isolectin B4, MSC, mesenchymal stem cell, PBS, phosphate-buffered saline, PS, penicillin/streptomycin, RT, room temperature, RV, right ventricular, RVOT, right ventricular outflow tract, RVOT-MS, fractional area of change in the right ventricular outflow tract, SIS-ECM, small intestinal submucosa–derived extracellular matrix, T-MSC, thymus-derived mesenchymal stem cell, VMSC, vascular smooth muscle cell

## Abstract

Graft cellularization holds great promise in overcoming the limitations associated with prosthetic materials currently used in corrective cardiac surgery. In this study, the authors evaluated the advantages of graft cellularization for right ventricular outflow tract reconstruction in a novel porcine model. After 4.5 months from implantation, improved myocardial strain, better endothelialization and cardiomyocyte incorporation, and reduced fibrosis were observed in the cellularized grafts compared with the acellular grafts. To the authors’ knowledge, this is the first demonstration of successful right ventricular outflow tract correction using bioengineered grafts in a large animal model.

## Highlights

• T-MSCs were isolated from the thymus gland of new born pigs, expanded, characterized and seeded onto a commercially available scaffold.
• The seeded-grafts were cultured within a bioreactor and then used to reconstruct the RVOT of a growing swine model.
• Pigs were followed up for 4.5 months; then scanned with a cardiac magnetic resonance and terminated to harvest the implants.
• By comparing the outcome of the seeded-grafts to the unseeded-ones used as control, we observed a reduced fibrosis and an improved RVOT strain, cardiac remodeling and endothelialization.

## Visual Abstract

**Figure:**
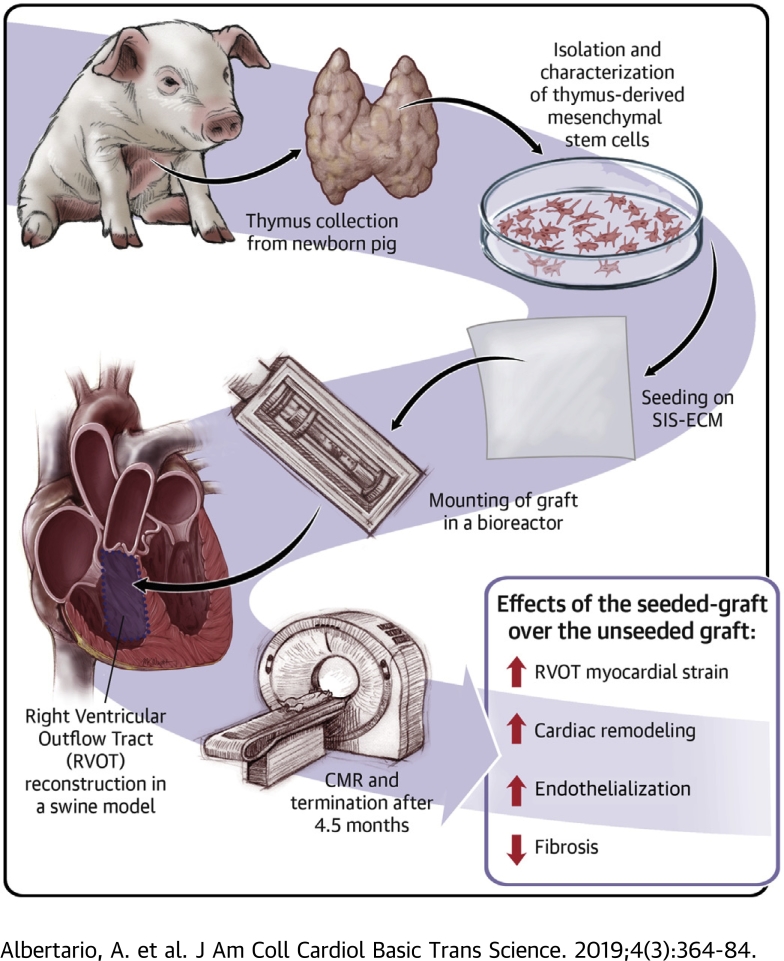

Cardiac tissue engineering holds great promise for definitive correction of congenital heart disease. By seeding cells on a biodegradable scaffold, this approach is aimed at developing a viable graft capable of growing and remodeling in parallel with the recipient’s organ. Once implanted in vivo, an ideal biofunctional graft is remodeled and eventually replaced by the host’s own extracellular matrix (1). Several cell types have been used for cardiac tissue engineering, including mesenchymal stem cells (MSCs) (2), induced pluripotent stem cells (3), endothelial cells (ECs) (4), and pericytes (5). In particular, MSCs are a preferred choice because of their immune-privileged nature, multilineage differentiation potential, and ability to promote tissue healing by paracrine mechanisms 6, 7. Furthermore, MSCs can be isolated from various tissues and organs, including bone marrow, umbilical cord, adipose tissue, and thymus 8, 9. Infants who undergo palliative cardiac surgery for tetralogy of Fallot often have the thymus removed to facilitate access to the heart. Hence, the explanted gland represents a convenient source of autologous MSCs for use during definitive correction of congenital cardiac defects.

To date, cardiac tissue engineering has been applied principally to the reconstruction of pulmonary arteries or valves, whereas problems associated with the right ventricular outflow tract (RVOT) remain an unmet clinical need. In fact, surgical correction of RVOT obstruction is reportedly associated with the development of aneurysmal or akinetic regions and an arrhythmogenic substrate (10). These complications have long-term consequences for outcomes in patients with tetralogy of Fallot (11).

In this paper, we report a controlled, randomized study in growing female piglets, comparing the feasibility, safety, and efficacy of RVOT reconstruction using a graft made of small intestinal submucosa–derived extracellular matrix (SIS-ECM) or the same graft engineered using porcine thymus-derived MSCs (T-MSCs).

## Methods

### Animals

Animals were treated in accordance with the Guide for the Care and Use of Laboratory Animals published by the National Institutes of Health in 1996 and conforming to the Animals (Scientific Procedures) Act of 1986. In vivo graft implantation was carried out under United Kingdom Home Office project license PPL 30/3019.

### Isolation and expansion of T-MSCs

T-MSCs were isolated from newborn female piglets as previously described (2). Briefly, cells were mechanically and enzymatically digested with collagenase I for 2 h at 37°C. The isolated cells were selected by adherence to plastic and expanded until passages 3 to 5.

### Neonatal rat cardiomyocyte isolation

Cardiomyocytes (CMs) were isolated from 1- to 3-day-old Wistar rats (Charles River Laboratories, Wilmington, Massachusetts). Three litters of neonatal rats were killed by means of decapitation, according to schedule 1 of the Animals (Scientific Procedures) Act. The hearts were washed with 1% penicillin/streptomycin (P/S) (Life Technologies, Carlsbad, California) phosphate-buffered saline (PBS) (Life Technologies), minced into thin pieces, and dissociated with 0.05% trypsin and 0.02% ethylenediaminetetraacetic acid in PBS for 15 min at 37°C. Cell suspension was filtered using a 70-μm nylon mesh, and enzyme activity was stopped with fetal bovine serum (FBS; Thermo Fisher Scientific, Waltham, Massachusetts). A second digestion was performed with 0.1% trypsin and 0.02% ethylenediaminetetraacetic acid in PBS for 15 min at 37°C, after which the cell fraction was filtered and the enzyme blocked with FBS. This procedure was repeated 3 more times. Then the cell fractions were pooled together and centrifuged for 5 min at 100*g*. The resulting pellet was resuspended in low-glucose Dulbecco’s modified Eagle’s medium (DMEM; Life Technologies), seeded onto a T75 flask and incubated for 1 h in a humidified chamber at 37°C in 5% CO_2_ (incubator) to allow as many fibroblasts as possible to attach to the plastic. The cell suspension was then harvested, centrifuged, and resuspended into 1% FBS and 1% P/S DMEM:M199 medium (Thermo Fisher Scientific) in a ratio of 1:1 and seeded onto previously coated plates with 0.1% gelatin (Sigma-Aldrich, St. Louis, Missouri).

### Flow cytometry

Fluorescence-activated cell sorting analysis was used to determine cell surface marker expression. The protocol was performed as described by Iacobazzi et al. (2). The following primary antibodies were used: 1:10 CD31-PE (Bio-Rad, Hercules, California), 1:600 CD44-APC (Thermo Fisher Scientific), 1:25 CD45-FITC (Bio-Rad), 1:10 CD73-APC (R and D Systems, Minneapolis, Minnesota), 1:20 CD90-PE (BioLegend, San Diego, California), and 1:5 CD105-PE (LSBio, Seattle, Washington). Analysis was performed on a NovoCyte flow cytometer (ACEA Bioscience, San Diego, California) using NovoExpress (ACEA Bioscience) for data collection and FlowJo (TreeStar, Ashland, Ohio) for analysis.

### In vitro multilineage differentiation

Multilineage differentiation into osteocytes, adipocytes, and chondrocytes was performed with MSCs at passages between 3 and 5. Cells were cultured in alpha-MEM medium with specific StemXVivo supplement kits (R and D Systems) for different time points. Osteogenic differentiation was assessed after 3 weeks of culture using alizarin red (Sigma-Aldrich) to detect calcium deposition. Oil red O staining (Sigma-Aldrich) was used to detect lipid accumulation of cells undergoing 2 weeks of adipogenic differentiation. Alcian blue staining (Sigma-Aldrich) was used to determine chondrogenic cartilage formation after 3 weeks of cell culture.

### SIS-ECM cellularization

Pieces of SIS-ECM (CorMatrix Cardiovascular, Roswell, Georgia) approximately 7 cm^2^ were soaked in DMEM for 24 h in a 1% agarose (Sigma-Aldrich) coated plate. The scaffold was then seeded with P3 swine T-MSCs at a density of 5 × 10^5^ cells/cm^2^ and cultured in growing medium for 1 week under static conditions in an incubator. The graft was then stitched to the rotating arm of a InBreath bioreactor (Harvard Apparatus, Cambridge, Massachusetts) and stitched back to itself as to fashion a conduit shape with the cells facing the outer side of the scaffold. The bioreactor chamber was filled with growing medium and connected to a motor platform placed at 37°C in a CO_2_ incubator. The rotation was initially set at 0.5 rpm and slowly increased within 24 h to the final speed of 2 rpm, which was maintained for 1 week.

### Cell viability

To determine cell viability on the engineered scaffolds, a Live/Dead Viability/Cytotoxicity Kit for mammalian cells (Thermo Fisher Scientific) was used according to the manufacturer’s instructions. Fluorescence imaging was carried out using a Zeiss Axio Observer.Z1 with Zen Blue software (Carl Zeiss, Oberkochen, Germany).

### Mechanical testing

Unseeded and seeded SIS-ECM pieces were analyzed for mechanical properties using an Instron 3343B machine (Instron, Norwood, Massachusetts) with pneumatic grips and a 100-N load cell. Crosshead speed was set at 10 mm/min. Samples were measured for tensile stress at maximum load and Young’s modulus using Bluehill software (Instron).

### Scanning electron microscopy

Samples were fixed, washed, and completely dehydrated as previously described (2). Surface topography was imaged using a Quanta 200 FEI field emission scanning electron microscope (Thermo Fisher Scientific).

For cell confluence quantification, ImageJ (National Institutes of Health, Bethesda, Maryland) was used on 1,000× images to measure the gaps between cells. This area was converted into a proportion and expressed as confluence rather then emptiness.

### In vivo studies

A total of 13 female Landrace piglets weighing 20 to 25 kg underwent cardiac surgery. Four additional unoperated pigs weighing approximately 90 kg were used as internal controls. All surgical procedures were performed under general anesthesia (ketamine, midazolam, dexmedetomidine, and isoflurane) and neuromuscular blockade (pancuronium bromide). The heart was exposed through a median sternotomy, and cardiopulmonary bypass was established by cannulating the inferior and superior vena cavae and the ascending aorta. An incision approximately 4 cm in length was made over the RVOT and below the pulmonary valve annulus, leaving the valve intact. The cut created was then patched with either regular SIS-ECM or T-MSC-seeded SIS-ECM. The latter was implanted with seeded cells facing the inner side of the heart. Ten swine were randomized to treatment according to a controlled study design. Details of the operation are reported in Supplemental Video 1. Animals were allowed to recover under continuous post-operative monitoring for the initial 24 to 48 h. Analgesic agents (paracetamol, morphine) and an antibiotic agent (cefuroxime) were administered regularly during this period.

### Doppler echocardiography

A 2-dimensional echocardiographic assessment was performed under general anesthesia using a 2-dimensional system (VividQ, GE Healthcare, Little Chalfont, United Kingdom) before graft implantation and 1, 2, 3, and 4.5 months thereafter.

### Cardiac magnetic resonance

Cardiac magnetic resonance was performed under anesthesia using a 3-T scanner (Siemens Healthcare, Erlangen, Germany). Dedicated long-axis and short-axis cine imaging of the RVOT was performed using steady-state free precession sequences. Biomechanical properties in the RVOT were investigated by measuring the fractional area of change in the RVOT (RVOT-MS) defined as follows: RVOT MS = [RVOT short-axis area (diastole) − RVOT short-axis area (systole)]/RVOT short-axis area (diastole).

### In vivo endpoints

The average in vivo follow-up of 10 of the operated animals was 145 ± 14 days, corresponding to approximately 4.5 months. The remaining 3 animals, all of them implanted with T-MSC SIS-ECM, were terminated 24 h, 1 week, and 2 weeks after implantation to determine the persistence of seeded cells in the graft post-surgery. Euthanasia was performed with an intravenous injection of 150 mg/kg of pentobarbital sodium.

### Histology

Explanted samples were washed in PBS and fixed overnight with 4% paraformaldehyde at 4°C. Fixed tissues were processed in a Thermo Excelsior AS (Thermo Fisher Scientific) and embedded with a Thermo HistoStar (Thermo Fisher Scientific) machine. Five-micrometer-thick sections were cut using a Shandon Finesse 325 microtome (Thermo Fisher Scientific). Hematoxylin and eosin, elastic van Gieson, and Masson’s trichrome staining was performed either manually or using a Shandon Varistain 24-4 (Thermo Fisher Scientific) automated machine. Von Kossa staining was carried out using a Silver plating kit (In Vitro Diagnostic Medical Device, Darmstadt, Germany) for the detection of microcalcification. Fibrosis was evaluated by measuring the collagen content of the explants in the Masson’s trichrome staining. Results were expressed as proportion of area occupied by collagen within the graft tissue.

### Fluorescent immunohistochemistry

Paraffin-embedded sections were deparaffinized by 2 changes of clearene and rehydrated through an alcohol gradient. A heated antigen retrieval with 10 mmol/l citrate buffer (pH 6.0) was performed. Samples were blocked with 10% goat serum (Sigma-Aldrich) in PBS for 30 min at room temperature (RT) and incubated with the unconjugated primary antibodies overnight at 4°C. A list of the primary and secondary antibodies is provided in Table 1. Fluorophore-conjugated secondary antibodies were incubated on the sections for 1 h at RT in the dark. Nuclei were counterstained with 4′,6-diamidino-2-phenylindole (1:1,000, Sigma-Aldrich) for 10 min at RT. Slides were mounted with Vectashield Hardset Mounting Medium (Vector Laboratories, Burlingame, California). Images were taken using a Zeiss Observer.Z1 fluorescent microscope.

**Table 1:** Antibodies for Immunohistochemistry

Primary Antibody	Primary Antibody Dilution	Manufacturer	Secondary Antibody	Secondary Antibody Dilution	Manufacturer
Mouse anti–alpha–sarcomeric actinin	1:100	Abcam	Goat antimouse–Alexa Fluor 488	1:400	Abcam
Mouse anti–alpha–smooth muscle actin	1:200	Abcam	Goat antimouse–Alexa Fluor 488	1:400	Abcam
Mouse anti–cardiac myosin heavy chain	1:150	Thermo Fisher Scientific	Goat antimouse–Alexa Fluor 488	1:400	Abcam
Rabbit anti–connexin-43	1:100	Santa Cruz Biotechnology	Goat antirabbit–Alexa Fluor 546	1:400	Abcam
Mouse anti–discoidin domain receptor 2	1:100	Santa Cruz Biotechnology	Goat antimouse–Alexa Fluor 488	1:400	Abcam
Isolectin B4-biotin	1:100	Life Technologies	Streptavidin–Alexa Fluor 488	1:200	Life Technologies
Rabbit anti-Ki67	1:200	Abcam	Goat antirabbit–Alexa Fluor 546	1:400	Abcam
Mouse anti–metalloproteinase 1	1:200	Abcam	Goat antimouse–Alexa Fluor 488	1:400	Abcam
Mouse anti–smooth muscle myosin heavy chain	1:100	Dako	Goat antimouse-Cy3	1:300	Jackson ImmunoResearch Laboratories

Primary antibodies used for immunofluorescent staining and respective secondary antibodies used for detection.

For microvasculature quantification, 10 random fields per section of the explanted grafts were analyzed under 20× magnification of a fluorescent microscope. Arterioles were quantified by counting the number of α–smooth muscle actin–positive vessels costained with isolectin B4 (IsoB4). Moreover, the number of α–smooth muscle actin–negative, IsoB4-positive vessels was counted to determine the number of capillaries developed in the healing cardiac tissue. The average number of arterioles and capillaries was then divided by the total acquired section area to calculate vascular density.

The graft regions composed of myocyte pockets were identified immunohistochemically using cardiac myosin heavy chain (cMYH) expression. Under a 20× magnification lens, 10 to 20 images were captured to cover all the cMYH-positive areas that developed in the newly formed tissue. Following identification of the regions containing CMs, ImageJ software was used to quantify the remodeling of the tissue sections. The images were split into individual channels. The ImageJ function threshold was applied in the channel of interest to convert each image to a binary version, where pixels were classified as representing either a region of myocytes or a region not containing myocytes. The pixels representing myocyte regions were quantified, pooled together, and converted into square millimeters. The myocyte area was then divided by the total area of the newly formed tissue. Similarly, the expression of the remodeling marker matrix metalloproteinase 1, the fibrotic protein discoidin domain-containing receptor 2, and the proliferation marker Ki67 were quantified using the same analytic method. Moreover, the isolectin-stained samples were used to measure the thickness of the endocardium that developed in the graft tissue of the seeded and unseeded patches after 4.5 months in vivo. The endocardium thickness of the native tissue was used as a positive control.

### Fluorescent in situ hybridization

Paraffin-embedded tissues were deparaffinized, rehydrated, and allowed to air-dry. Slides were incubated with 30% sodium bisulfite (Sigma-Aldrich) for 20 min at 37°C and washed with 2× SSC (Thermo Fisher Scientific). Samples were incubated with a proteinase K (Qiagen, Hilden, Germany) solution for 15 min at 45°C and then washed with 2× SSC. Slides were rehydrated through an alcohol gradient and allowed to air-dry. The Y-chromosome probe mixture (Chrombios, Nussdorf AM Inn, Germany) was added to the samples. The slides were placed on a hotplate for 10 min at 80°C and then transferred overnight to a humidified chamber at 37°C. At the end of the hybridization, the samples were washed with 0.4× SSC at 73°C and with 2× SSC at RT. Nuclei were counterstained with 4′,6-diamidino-2-phenylindole (1:1,000) and mounted with Vectashield Hardset Mounting Medium. Fluorescent in situ hybridization was performed on the seeded grafts before in vivo implantation and on seeded grafts explanted after 24 h, 1 week, 2 weeks, and 4.5 months. The number of cells expressing the Y chromosome was measured in the seeded grafts before implantation and after the defined time points. Results were expressed as density of Y chromosome–positive cells per square millimeter.

### Effects of T-MSCs on CMs

The in vitro proliferation and apoptosis of P0 rat CMs primary culture were measured respectively with Cell Proliferation ELISA, BrdU (Roche, Basel, Switzerland) and Caspase-Glo 3/7 Assay (Promega, Madison, Wisconsin). The CMs were either cultured with the T-MSC-conditioned medium or cocultured with the T-MSCs. Three independent experiments were carried out. The conditioned medium was freshly harvested from passage 2 and 3 T-MSCs, centrifuged, mixed with 1% FBS and 1% P/S M199 at a ratio of 1:1, and added to the CMs. Regarding the coculture condition, P0 primary culture of rat CMs was seeded in 96-well plates, onto which cell culture inserts (CellCrown inserts, Sigma-Aldrich) were placed and seeded with T-MSCs. CMs cultured with 1% FBS and 1% P/S DMEM:M199 (1:1) were used as a negative control. Twenty-four hours after seeding, bromodeoxyuridine was added to the cells to assess proliferation. Cells were incubated for 24 h at 37°C in a CO_2_ incubator, and the enzyme-linked immunosorbent assay kit was used according to the manufacturer’s instructions. Absorbance was measured at 450 nm using a microplate photometer (Opsys MR, Dynex Technologies, Chantilly, Virginia). Apoptosis was measured 48 h after seeding. Caspase reagent was incubated with the cells for 45 min at RT. The resulting luminescence was detected at 485 nm by using a microplate luminometer (GloMax, Promega).

A migration assay was performed on CMs to investigate the effect of the T-MSCs on the migration potential of the cardiac cells. CMs were either cultured with the T-MSC-conditioned medium or cocultured with the stem cells in a 24-well plate. After confluence was reached, the cell monolayer was scratched with a P200 tip at the center of the well. Then the cells were washed and 2 mmol/l hydroxyurea (Sigma-Aldrich) was added to the media. Images were taken using a bright-field microscope at 4×. The proportion of gap closure was measured using ImageJ to calculate the area of the wound at the time of the scratch and 14 h later. Each experiment was performed in triplicate and repeated 3 times.

Released cytokines and growth factors were analyzed in the secretome of T-MSCs. A customized cytokine/chemokine multiplex kit (Millipore, Burlington, Massachusetts) composed of magnetic beads for the detection of interleukin (IL)–1ra, IL-2, IL-6, IL-8, interferon-γ, and tumor necrosis factor–α was used according to the manufacturer’s instructions. The analysis was limited to 6 analytes because of the low availability of porcine antibodies. Reading was performed using a Luminex MAGPIX instrument (Luminex, Austin, Texas). Standard culture medium was used as control for data normalization.

### Statistical analysis

Data are expressed as mean ± SEM. Statistical analysis was carried out using the Student's t-test to determine the difference between 2 groups. Alternatively, 1-way analysis of variance followed by Tukey post hoc testing was applied to analyze the differences among multiple groups. Results were considered statistically significant at p < 0.05.

## Results

### Characterization of T-MSCs

**Figure 1:**
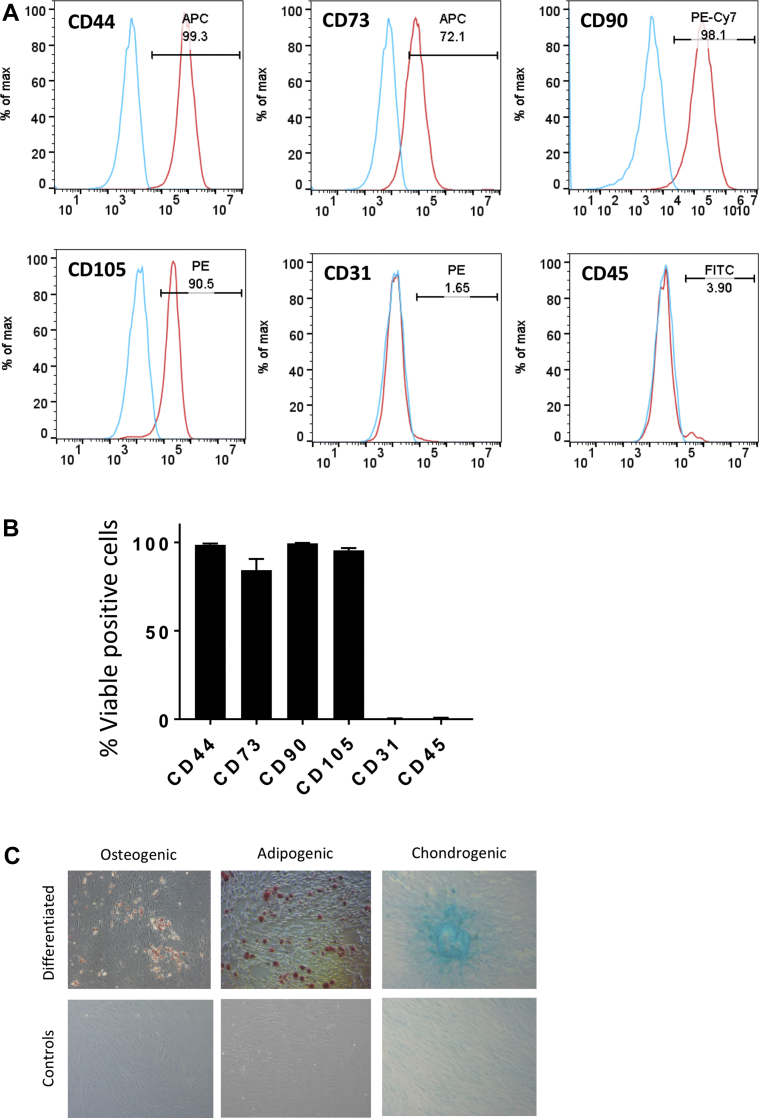
Phenotypic and Functional Characterization of Thymus-Derived Mesenchymal Stem Cells **(A)** Flow cytometry representative histograms illustrate the expression of CD44, CD73, CD90, and CD105 and lack of endothelial and hematopoietic markers on thymus-derived mesenchymal stem cells. **(B)** Bar chart showing the proportion of viable cells positive to the investigated markers (n = 4; mean ± SE). **(C)** Multilineage differentiation of the cells into osteocytes, adipocytes. Nontreated cells were used as negative controls.

Adherent cells from the swine thymus gland exhibited the typical spindle-shape morphology. Flow cytometry of 4 cell lines showed abundant expression of the mesenchymal markers CD44, CD73, CD90, and CD105 and negativity for CD31 and CD45 (Figures 1A and 1B). Furthermore, we confirmed the ability of T-MSCs to differentiate into mesenchymal lineages (Figure 1C).

### Characterization of T-MSC-engineered SIS-ECM scaffolds

**Figure 2:**
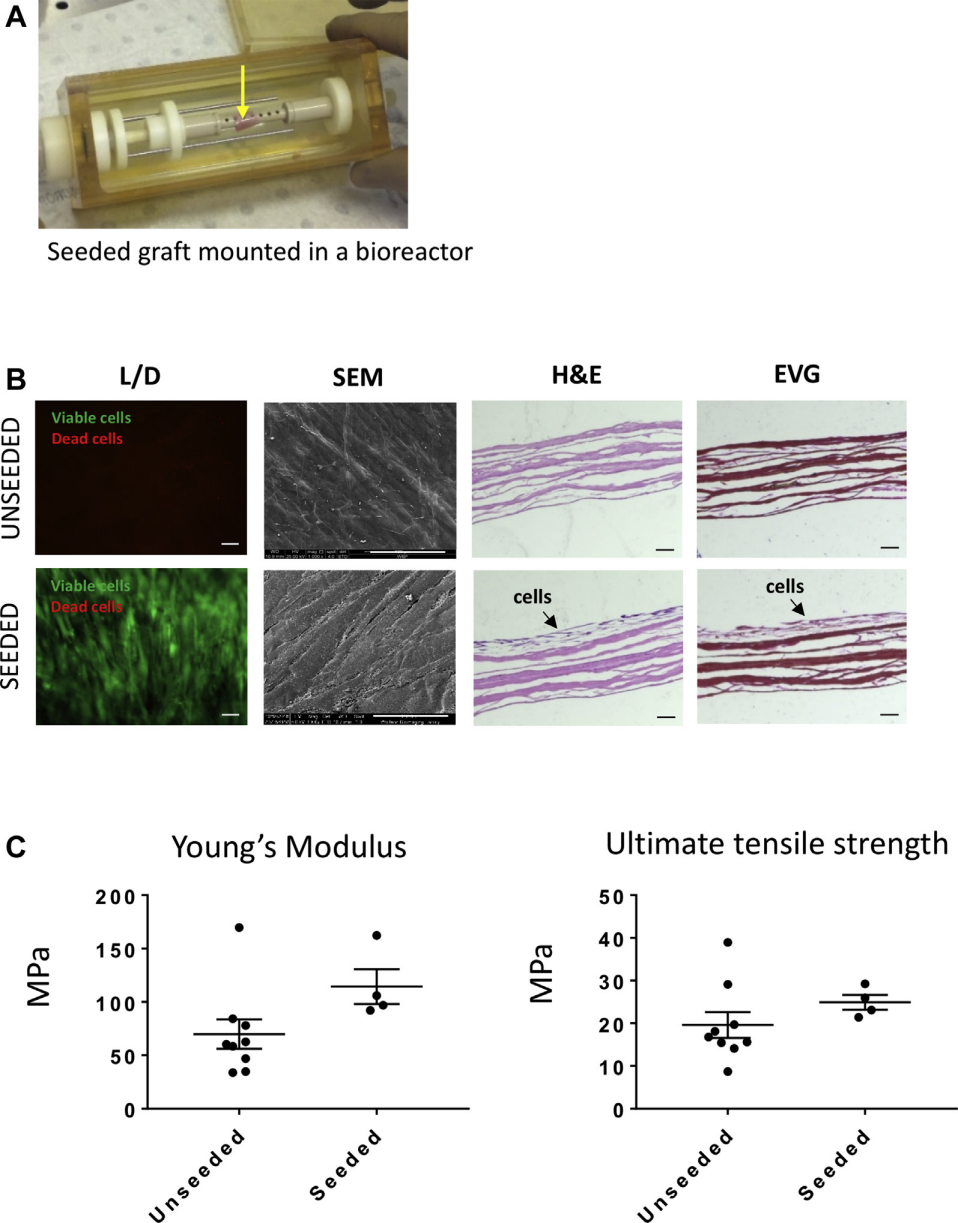
Biological and Mechanical Assessment of the Unseeded and Cell-Seeded Grafts **(A)** Seeded graft **(arrow)** is mounted and cultured in a bioreactor. **(B)** Viable (L) and dead (D) cells attached to the seeded scaffold. Scanning electron microscopic (SEM) images illustrate the topography of the unseeded graft and a confluent layer of oriented cells growing on the seeded sample. Scale bars, 100 μm. Hematoxylin and eosin (H and E) staining shows lack of nuclei in the unseeded scaffold and a multilayer of cells in the seeded graft. Elastic van Gieson (EVG) staining confirmed the capacity of the seeded cells to produce their own extracellular matrix. Scale bars, 50 μm. **(C)** Young’s modulus and ultimate tensile strength of the unseeded and seeded grafts showed no significant differences between the 2 groups (n = 4; mean ± SE).

The T-MSCs seeded on the SIS-ECM scaffold were grown for 1 week under static condition and then 1 week under dynamic condition in a bioreactor (Figure 2A). T-MSCs were viable within the scaffold at 2 weeks from seeding, with only a few dead cells being observed (4.0 ± 0.6% of total cell counts) (Figure 2B). They were stratified on the surface of the scaffold (hematoxylin and eosin staining) and interposed with newly formed collagen (elastic van Gieson staining). Scanning electron microscopy confirmed the presence of a confluent and oriented layer of cells on the surface of the SIS-ECM scaffold.

Next, we compared the mechanical characteristics of the unseeded and T-MSC-seeded scaffolds. No difference between groups was observed with regard to stiffness or tensile stress at maximum load (p = 0.1075 and p = 0.2890, respectively) (Figure 2C).

### Feasibility and safety of T-MSC-engineered grafts implantation

Figure 3A illustrates the topography (i) and operative characteristics of the RVOT lesion (ii–iv). All operated animals survived the surgical procedure and recovered well, with no loss during follow-up and similar body weight gain (Figure 3B). At termination, body weight was similar to that of unoperated swine of the same age (91 ± 6 kg and 93 ± 7 kg, respectively, in the unseeded and seeded groups vs. 90 ± 1 kg in control groups). Echocardiography showed similar values of RVOT diameters (control subject vs. unseeded; p = 0.1726; control subject vs. seeded; p = 0.995), left ventricular contractile function, and left ventricular volumes among unoperated, unseeded, and T-MSC-seeded groups (Figure 3C, Table 2). Likewise, Doppler analysis demonstrated no change in maximum RVOT velocity between the control subject and operated animals (control subject vs. unseeded; p = 0.1569; control subject vs. seeded; p = 0.052) (Figure 3C). Similarly, the proportion of right ventricular (RV) fractional area change did not differ between groups and was within clinical reference range (32% to 60%) of normal RV function (Table 2).Figure 3in Vivo Right Ventricular Outflow Tract Reconstruction and Operated Animals Follow-Up**(A)** Cartoon **(i)** and macroscopic image **(ii)** showing the site of the implant on the right ventricular outflow tract (RVOT). Gross analysis of the explants after 4.5 months in vivo shows the epicardial **(iii)** and endocardial **(iv)** sides of the graft **(dotted line)**. **(B)** The operated pigs increased their body weight at a normal rate. **(C)** Doppler echocardiographic measurements of the right ventricle immediately before termination demonstrate comparable RVOT diameter and maximum velocity (Vmax) in the operated animals and unoperated control (Ctr) pigs. **(D)** Representative cardiac magnetic resonance images of right ventricular diastolic and systolic area (encircled in **green**) before surgery and 4.5 months thereafter. The patches could be visualized as a small bump protruding from the right ventricle **(arrows)**. **(E)** RVOT myocardial strain measured at termination was greater in the animals implanted with seeded grafts. See Supplemental Video 1.
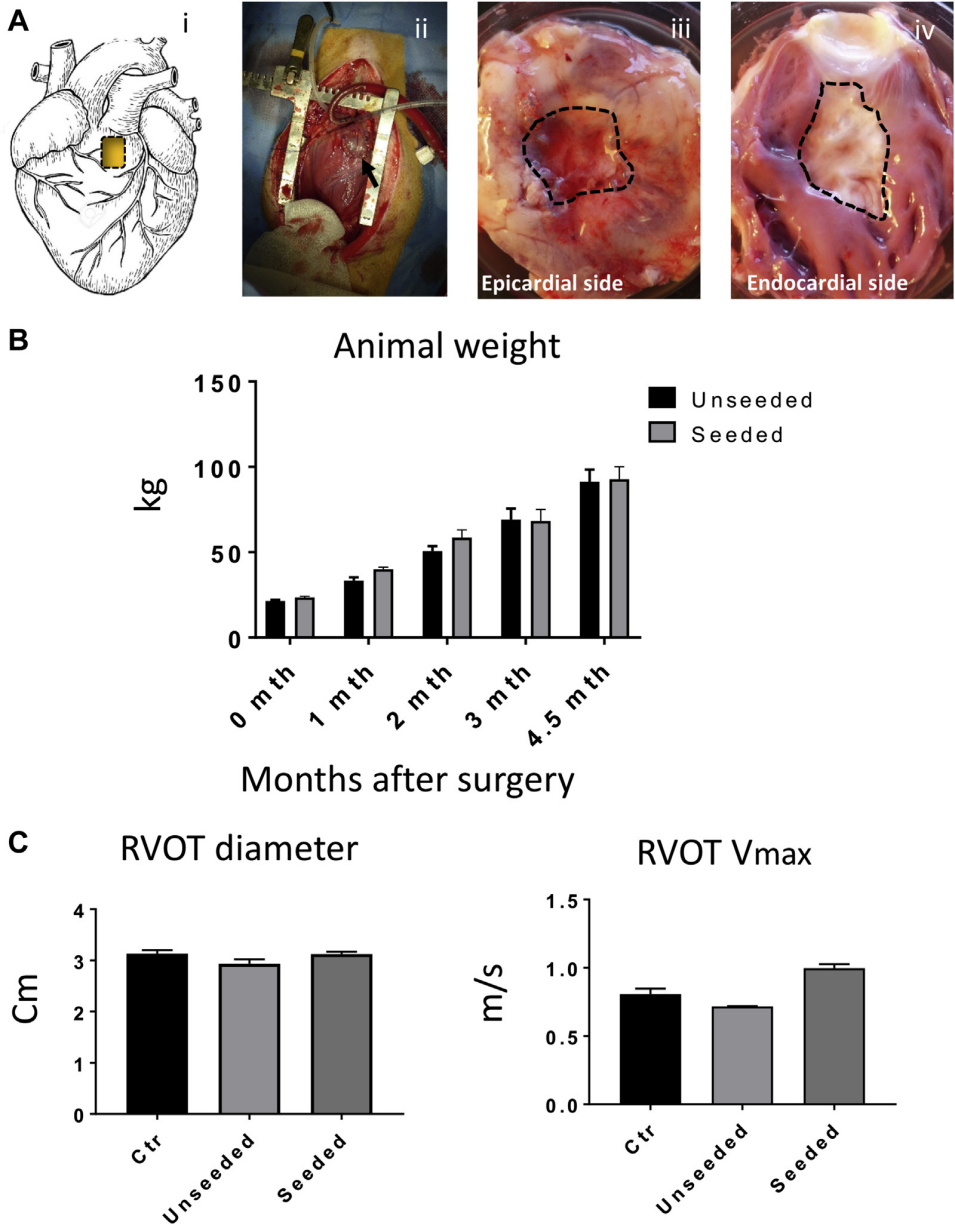

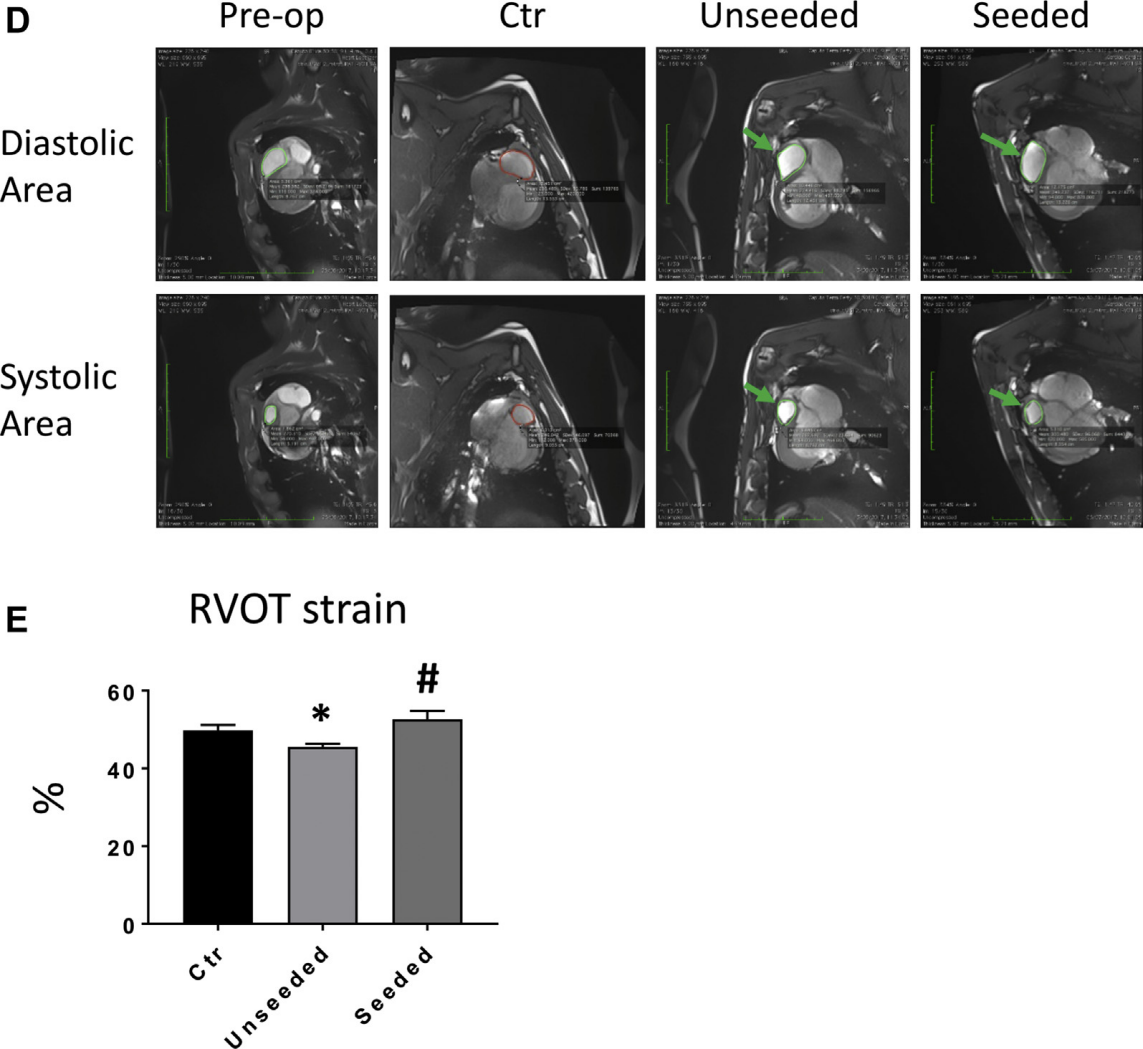

**Figure:**
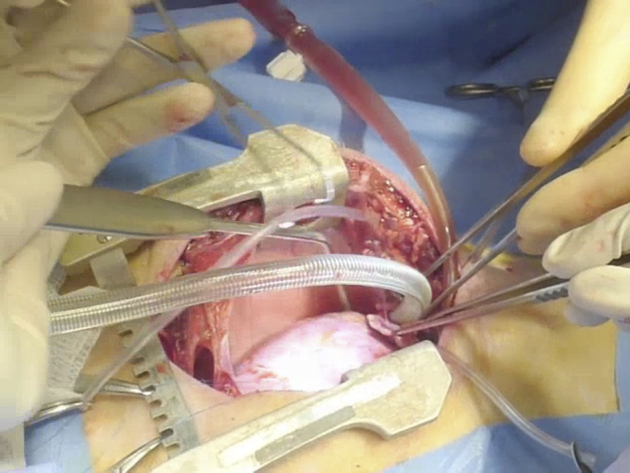
Supplemental Video 1

**Table 2:** Doppler Echocardiographic Measurements

	Pre-Operative	Control	Post-Operative Unseeded	Post-Operative Seeded
IVSd (cm)	1.12 ± 0.48	1.30 ± 0.10	1.40 ± 0.17	1.35 ± 0.16
IVSs (cm)	0.89 ± 0.08	2.10 ± 0.10	1.60 ± 0.17	2.00 ± 0.22
LVIDd (cm)	2.82 ± 0.12	3.40 ± 0.23	3.43 ± 0.46	3.72 ± 0.14
LVIDs (cm)	1.96 ± 0.13	1.75 ± 0.27	1.70 ± 0.44	2.10 ± 0.07
LVPWd (cm)	0.85 ± 0.06	1.20 ± 0.11	1.36 ± 0.20	1.62 ± 0.11
LVPWs (cm)	1.37 ± 0.10	2.25 ± 0.15	2.16 ± 0.03	2.15 ± 0.12
EDV (ml)	31 ± 3	49 ± 8	50 ± 15	59 ± 5
ESV (ml)	12 ± 2	11 ± 3	10 ± 6	14 ± 1
EF (%)	60 ± 5	80 ± 6	83 ± 6	76 ± 4
SV (ml)	18 ± 2	39 ± 6	40 ± 10	46 ± 6
FS (%)	31.3 ± 3.1	50.0 ± 6.6	51.3 ± 5.6	44.0 ± 3.9
RV FAC (%)	48.97 ± 5.41	50.80 ± 2.88	49.16 ± 2.89	47.11 ± 3.5

Values are mean ± SE. Left ventricular and RV function measured using electrocardiography before surgery and 4.5 months thereafter. Unoperated adult pigs were used as control subjects.

EDV = end-diastolic volume; EF = ejection fraction; ESV = end-systolic volume; FAC = fractional area change; FS = fractional shortening; IVSd = intraventricular septum in diastole; IVSs = interventricular septum in systole; LVIDd = left ventricular internal diameter in diastole; LVIDs = left ventricular internal diameter in systole; LVPWd = left ventricular posterior wall diastole; LVPWs = left ventricular posterior wall systole; RV = right ventricular; SV = stroke volume.

### Implantation of T-MSC-engineered grafts improves RVOT contractility compared with unseeded grafts

Cardiac cardiac magnetic resonance assessments of RVOT motion and deformation were carried out before surgery and at termination, using unoperated adult pigs as control subjects for the latter time point (Figure 3D). Basal RVOT-MS values did not differ between the study groups, thus excluding a chance of imbalance that may influence outcome, and were therefore cumulated (Table 3). At the final measurement, unseeded animals showed reduced RVOT-MS compared with unoperated control subjects (p < 0.05), with this gap being totally abrogated in the T-MSC-seeded group (control subject vs. unseeded; p = 0.1835; control subject vs. seeded; p = 0.0432; unseeded vs. seeded; p = 0.0408) (Figure 3E). The RVOT-MS increase observed in the seeded group was the consequence of a reduction of the systolic area, whereas diastolic area did not differ between the 2 groups (Table 3).

**Table 3:** Cardiac Magnetic Resonance Imaging Measurements

	Pre-Operative	Unoperated Controls	Post-Operative Unseeded	Post-Operative Seeded
Diastolic area (cm^2^)	3.91 ± 0.25	11.98 ± 0.56	11.87 ± 0.73	12.01 ± 0.10
Systolic area (cm^2^)	1.96 ± 0.22	6.03 ± 0.40	6.48 ± 0.45	5.68 ± 0.21
Strain (%)	51 ± 3	50 ± 1.08	45.50 ± 0.84∗	52.63 ± 2.17∗

Values are mean ± SE. Diastolic and systolic area and right ventricular outflow tract strain as assessed using cardiac cardiac magnetic resonance before surgery (pre-operative) and 4.5 months thereafter (unseeded and seeded animals). Unoperated adult pigs were used as control subjects.

∗ p < 0.05 when comparing unseeded control animals vs. unoperated control animals, and seeded vs. unseeded animals.

### In vivo integration and remodeling of T-MSC-engineered grafts within the host cardiac tissue

**Figure 4:**
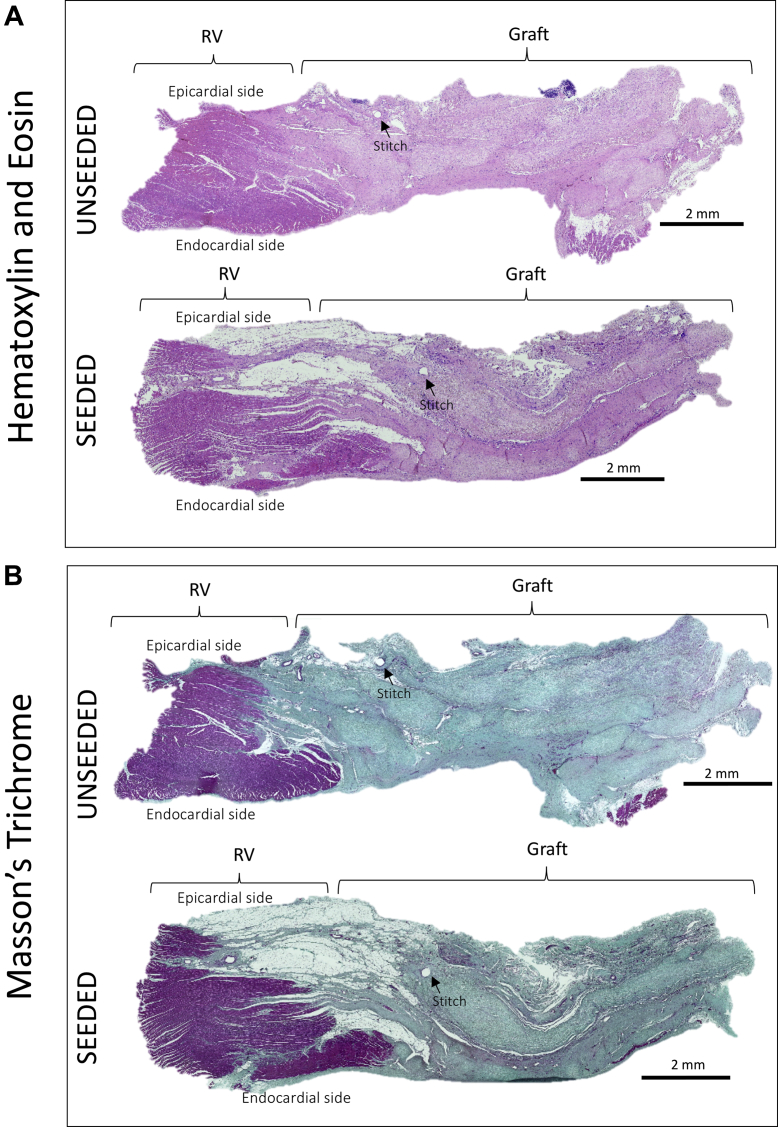

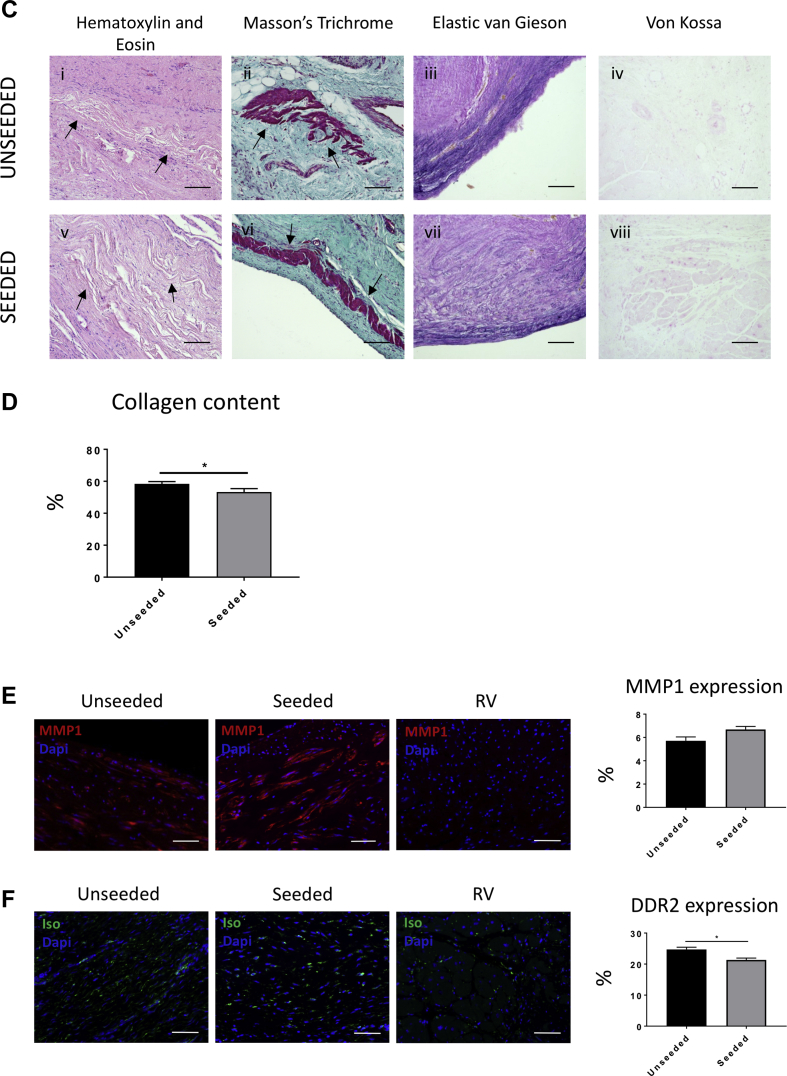

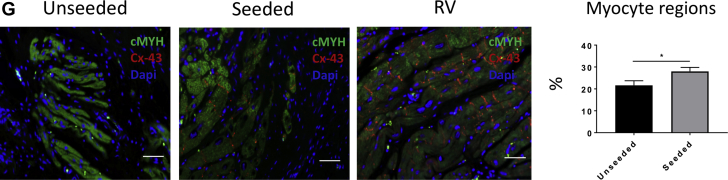
Examination of the Explanted Grafts **(A,B)** Hematoxylin and eosin (H and E) and Masson’s trichrome staining of the explants illustrates the collagen-rich grafts well integrated with the right ventricle (RV). **(C)** Higher magnification images of the samples. H and E staining shows little remains of the scaffold **(arrows)** 4.5 months after implantation **(i,v)**. Masson’s trichrome staining demonstrates the presence of new muscle tissue **(arrows)** generated from the implanted grafts **(ii,vi)**. Elastic van Gieson staining illustrates an elastin-rich endothelium laying the inner side of the explants **(iii,vii)**. No calcification was detected as shown by von Kossa stain **(iv,viii)**. **(D)** More abundance of collagen was detected in the unseeded samples in comparison with the seeded group. **(E)** Immunostaining for matrix metalloproteinase 1 (MMP1) confirmed that the extracellular matrix was undergoing remodeling processes, and no differences were observed between the 2 groups. **(F)** Immunostaining and quantification of the fibrotic marker discoidin domain-containing receptor 2 (DDR2) (**green** fluorescence) showed more fibrosis in the unseeded explants compared with the seeded ones. **(G)** Cells expressing cardiac myosin heavy chain (cMYH) were present in explants from both groups, though more abundant in the seeded one. In addition, only the seeded graft contained cells double positive for cMYH and connexin-43 (Cx-43). Scale bars, 50 μm. Dapi = 4′,6-diamidino-2-phenylindole; Iso = isolectin B4.

The results of cardiac cardiac magnetic resonance suggest that T-MSCs can confer the implanted scaffold with the capacity to preserve RVOT contractility. Therefore, we investigated the mechanisms responsible for this beneficial effect. At macroscopic inspection, explanted RVOTs presented a smooth luminal surface with no sign of tissue degradation in both groups. Hematoxylin and eosin staining showed in vivo integration of the grafts within the neighboring myocardium (Figure 4A). The newly formed tissue within the implants was composed mainly of collagenous fibers, as shown by Masson’s trichrome (Figure 4B) and elastic van Gieson (Supplemental Figure 1) staining. A close inspection revealed only few discernible remnants of the SIS-ECM scaffold (Figure 4C), suggesting that the implanted graft underwent remodeling and new tissue formation. Moreover, von Kossa staining showed no calcification in the explanted cell-seeded or unseeded grafts (Figure 4C, Supplemental Figure 2). Interestingly, the amount of collagen developed in the cell-seeded explants was significantly lower compared with the unseeded explants (53.31 ± 2.07% and 58.45 ± 1.36% of total section area, respectively; p = 0.0429) (Figure 4D). Immunostaining and quantification of the remodeling marker matrix metalloproteinase 1 demonstrated similar expression in the unseeded and seeded groups (5.91 ± 0.32% and 6.67 ± 0.25%, respectively; p = 0.0516) (Figure 4E). Moreover, discoidin domain-containing receptor 2 was significantly down-regulated in the cell-seeded compared with the unseeded group (22.5 ± 0.64% vs. 24.7 ± 0.73%, respectively; p = 0.0422) (Figure 4F), which, together with the data of collagen content, suggests an antifibrotic effect that was exerted by T-MSCs. The control RV tissue distant from the implant was negative for the matrix metalloproteinase 1 and discoidin domain-containing receptor 2 markers.

**Figure 5:**
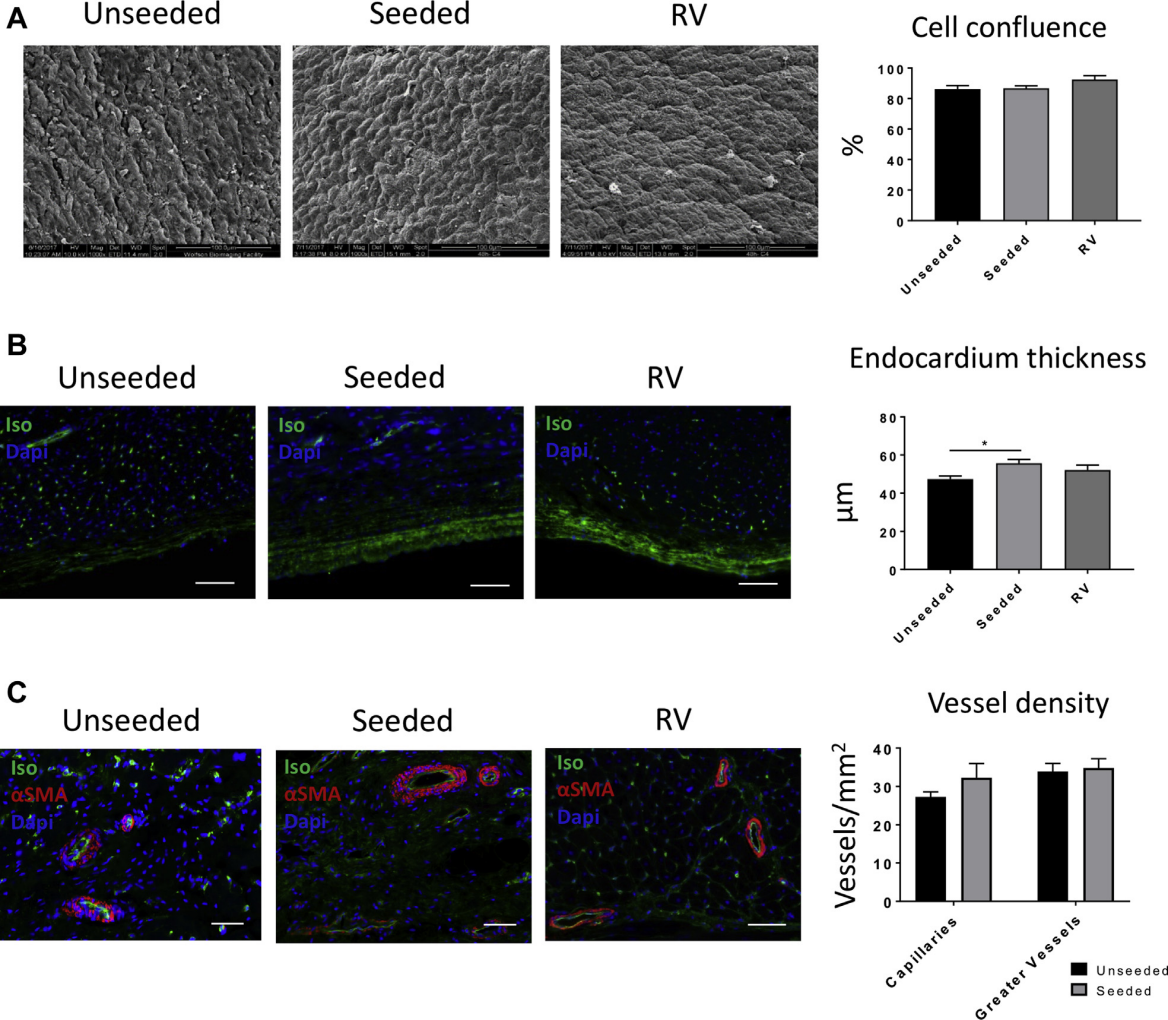
Evaluation of the Endothelialization and Vascularization of the Explants **(A)** Scanning electron microscopic images show that the topography of the endocardial surface of the explants is composed of aligned and compacted cells. Similar cell confluency to the native tissue was detected in the unseeded and seeded patches. **(B)** By measuring the thickness of the endocardium of the sections stained for isolectin B4 (Iso), we observed that the seeded group developed a thicker layer than the unseeded group **(C)**. The explanted grafts generated a fine and rich vascularization shown by isolectin B4 and alpha–smooth muscle actin (αSMA) staining. However, no differences were observed between the 2 groups in terms of capillaries and arteriole density. Scale bars, 50 μm. Dapi = 4′,6-diamidino-2-phenylindole; RV = right ventricle.

Immunostaining for the cardiac marker cMYH confirmed the presence of myocyte-like clusters dispersed within the explants of both unseeded and cell-seeded groups (Figure 4G). Interestingly, the area of the explant occupied by these myocyte-like islets was greater in the cell-seeded group (p = 0.0462). Moreover, seeded grafts contained myocytes that coexpress cMYH and the ventricular gap junction protein connexin-43 (Cx-43), whereas these double-positive myocytes were absent in unseeded grafts. Scanning electron microscopy of the endocardial side of the explanted grafts showed that both the unseeded and seeded patches developed a compact cell layer, resembling the topography of the native tissue (cell confluence 86.12 ± 2.39%, 86.77 ± 1.62%, and 92.54 ± 2.36%, respectively; unseeded vs. seeded; p = 0.9702; unseeded vs. RV; p = 0.1598; seeded vs. RV; p = 0.2045) (Figure 5A). Immunostaining with IsoB4 demonstrated that the seeded grafts developed a thicker layer of ECs compared with unseeded group (53.54 ± 2.04 and 46.45 ± 1.81 μm, respectively; unseeded vs. seeded; p = 0.0291; unseeded vs. RV; p = 0.2793; seeded vs. RV; p = 0.4611) (Figure 5B).

Fluorescent microscopy identified capillaries and arterioles within the grafts, although the former were less abundant than in control RV myocardium, suggesting that graft arteriogenesis prevailed on capillarization in the healing process of tissue (Figure 5C).

**Figure 6:**
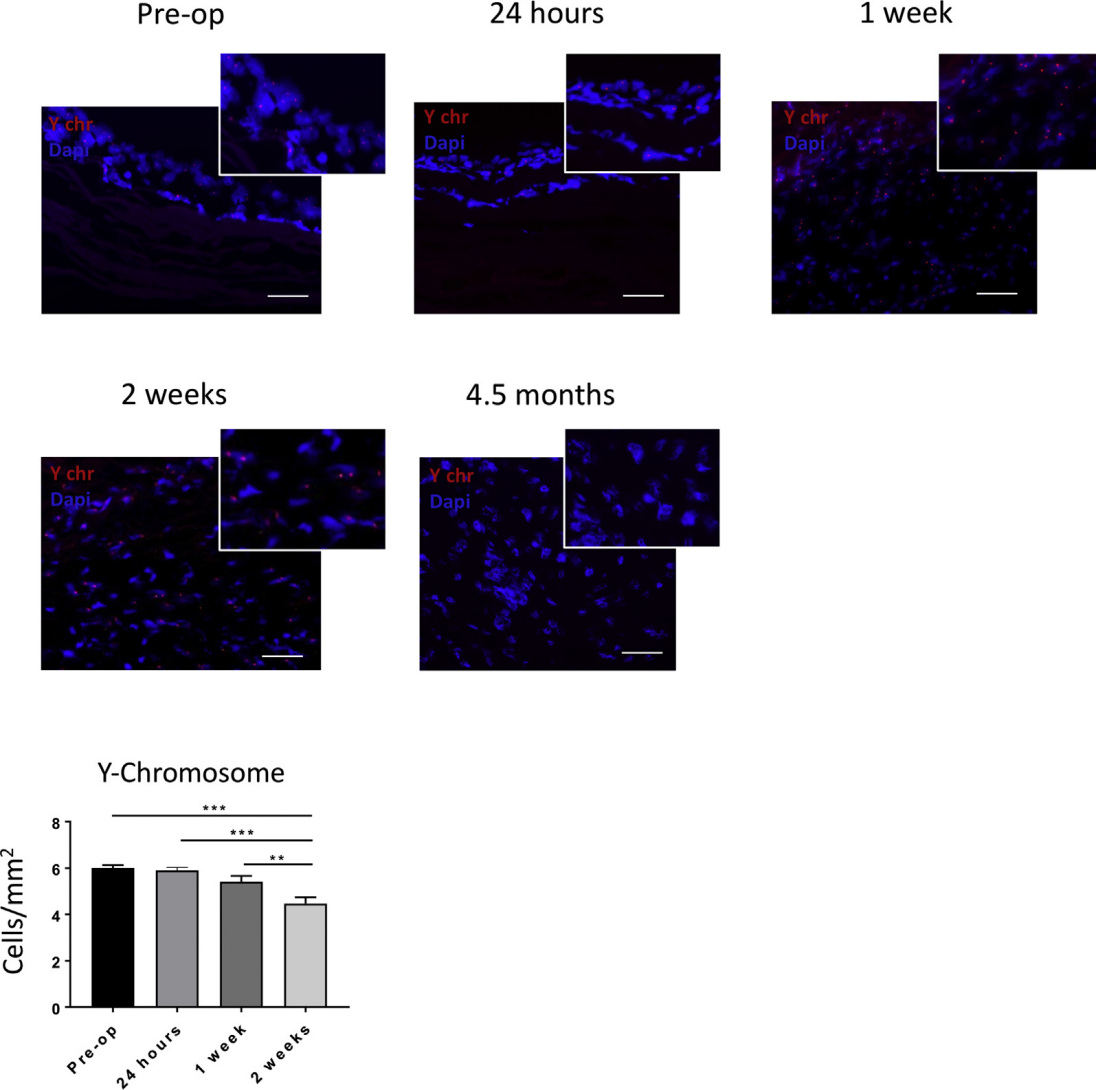
Y Chromosome Fluorescent in Situ Hybridization of the Explanted Grafts The male seeded cells (Y chromosome [Y chr] positive) were detected over the scaffold before implantation and after 24 h, 1 week, and 2 weeks. The number of seeded cells decreased with time in vivo. No seeded cells were observed at 4.5 months. Scale bars, 50 μm. Dapi = 4′,6-diamidino-2-phenylindole.

3The improved physiological remodeling in the cell-seeded group prompted us to examine the fate of the implanted male donor cells into the female recipients using fluorescent in situ hybridization analysis of Y chromosome in the explanted grafts. Cells expressing the Y chromosome were detected in grafts explanted 24 h, 1 week, and 2 weeks post-implantation (Figure 6). We observed a progressive decline in the abundance of cells expressing the Y chromosome, but positive cells were still present at 2 weeks after implantation (pre-operative vs. 24 h; p = 0.9842; pre-operative vs. 1 week, p = 0.2040; pre-operative vs. 2 weeks; p < 0.0001; 24 h vs. 1 week; p = 0.03695; 24 h vs. 2 weeks; p < 0.001; 1 week vs. 2 weeks; p = 0.009). In contrast, no Y chromosome–positive cells were detected at 4.5 months. Therefore, it is most likely that CMs and ECs found in the explanted grafts derive from the recipient rather than from donor T-MSCs.

**Figure 7:**
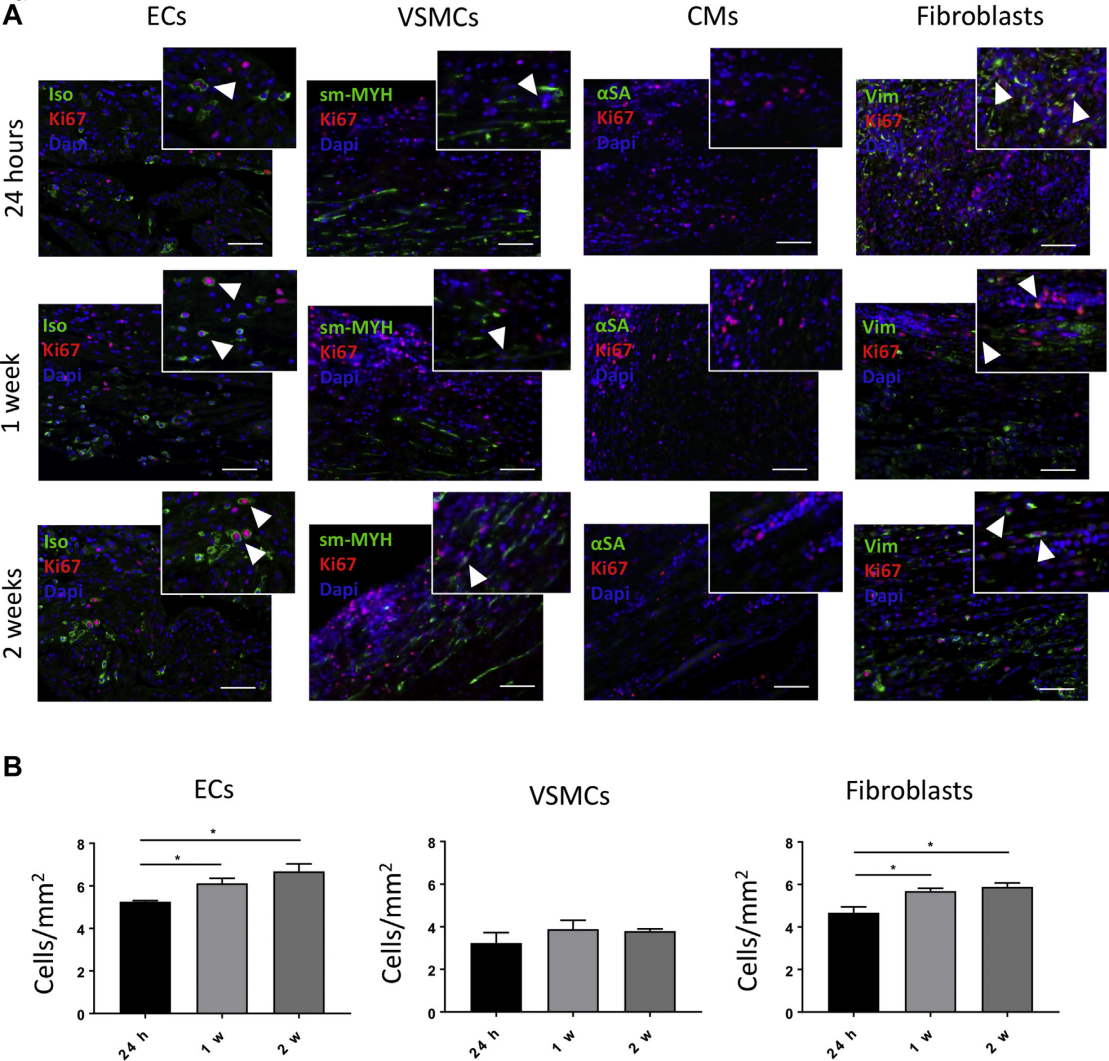

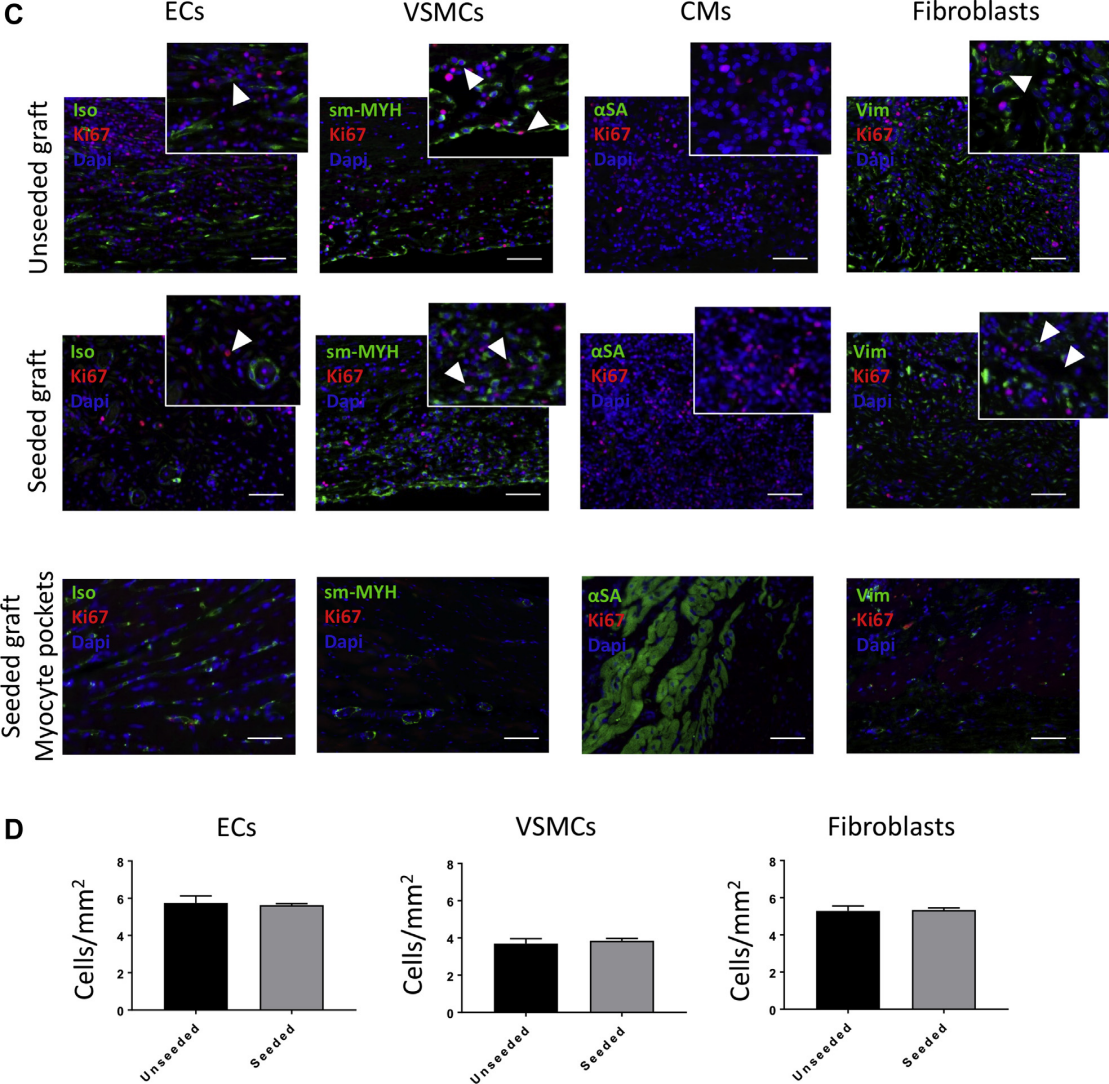
Evaluation of Proliferating Cells in Vivo **(A)** Immunostaining for Ki67 and isolectin B4 (Iso), smooth muscle myosin heavy chain (sm-MYH), α–sarcomeric actinin (αSA), and vimentin (Vim) in the seeded grafts explanted at 24 h, 1 week, and 2 weeks. Coexpression of Ki67 with Iso and Vim suggests that the proliferating cells at early stages consist of endothelial cells (ECs) and fibroblasts. Scale bars, 50 μm. **(B)** The numbers of proliferating ECs and fibroblasts increase after 1 week in vivo, with the fibroblasts showing an increased proliferation also after 2 weeks. **(C)** Double staining for Ki67 and Iso, sm-MYH, αSA, and Vim in the unseeded and seeded grafts explanted at 4.5 months. Cells coexpressing Ki67 with Iso, sm-MYH, and Vim were detected in the grafts, suggesting that the proliferating cells are ECs, vascular smooth muscle cells (VSMCs), and fibroblasts. Scale bars, 50 μm. **(D)** The number of Ki67-positive cells seemed to be the same between the unseeded and seeded groups 4.5 months after implantation.

Next, we measured cell proliferation in grafts explanted at 24 h, 1 week, 2 weeks, and 4.5 months by immunostaining for Ki67, in combination with IsoB4, smooth muscle myosin heavy chain, α–sarcomeric actinin, and vimentin to detect, respectively, proliferating ECs, vascular smooth muscle cells (VSMCs), CMs, and fibroblasts (Figure 7). Some Ki67-positive ECs and fibroblast-like cells were observed in the seeded grafts explanted after 24 h, 1 week, and 2 weeks, whereas neither VSMCs nor CMs were positive for Ki67 (Figure 7A). The number of proliferating ECs increased after 1 week; likewise fibroblasts showed greater proliferation at 1 and 2 weeks compared with 24 h (ECs: 24 h vs. 1 week; p = 0.0411; 24 h vs. 2 weeks; p = 0.0141; 1 week vs. 2 weeks; p = 0.3021; VSMCs: 24 h vs. 1 week; p = 0.4812; 24 h vs. 2 weeks, p = 0.5724; 1 week vs. 2 weeks; p = 0.9839; fibroblasts: 24 h vs. 1 week; p = 0.0284; 24 h vs. 2 weeks; p = 0.0126; 1 week vs. 2 weeks; p = 0.7590) (Figure 7B). Additionally, we analyzed the proliferating cells in the seeded and unseeded grafts explanted after 4.5 months (Figure 7C). Ki67-positive ECs, VSMCs, and fibroblasts were detected at this time point within the grafts, but no proliferating cells were found in the myocyte pockets, and no differences were observed regarding the number of proliferating ECs, VSMCs, and fibroblasts (ECs: unseeded vs. seeded; p = 0.7649; VSMCs: unseeded vs. seeded; p = 0.6262; fibroblasts: unseeded vs. seeded; p = 0.8648) (Figure 7D).

### Evaluation of the paracrine effect of the T-MSCs on the CMs in vitro

**Figure 8:**
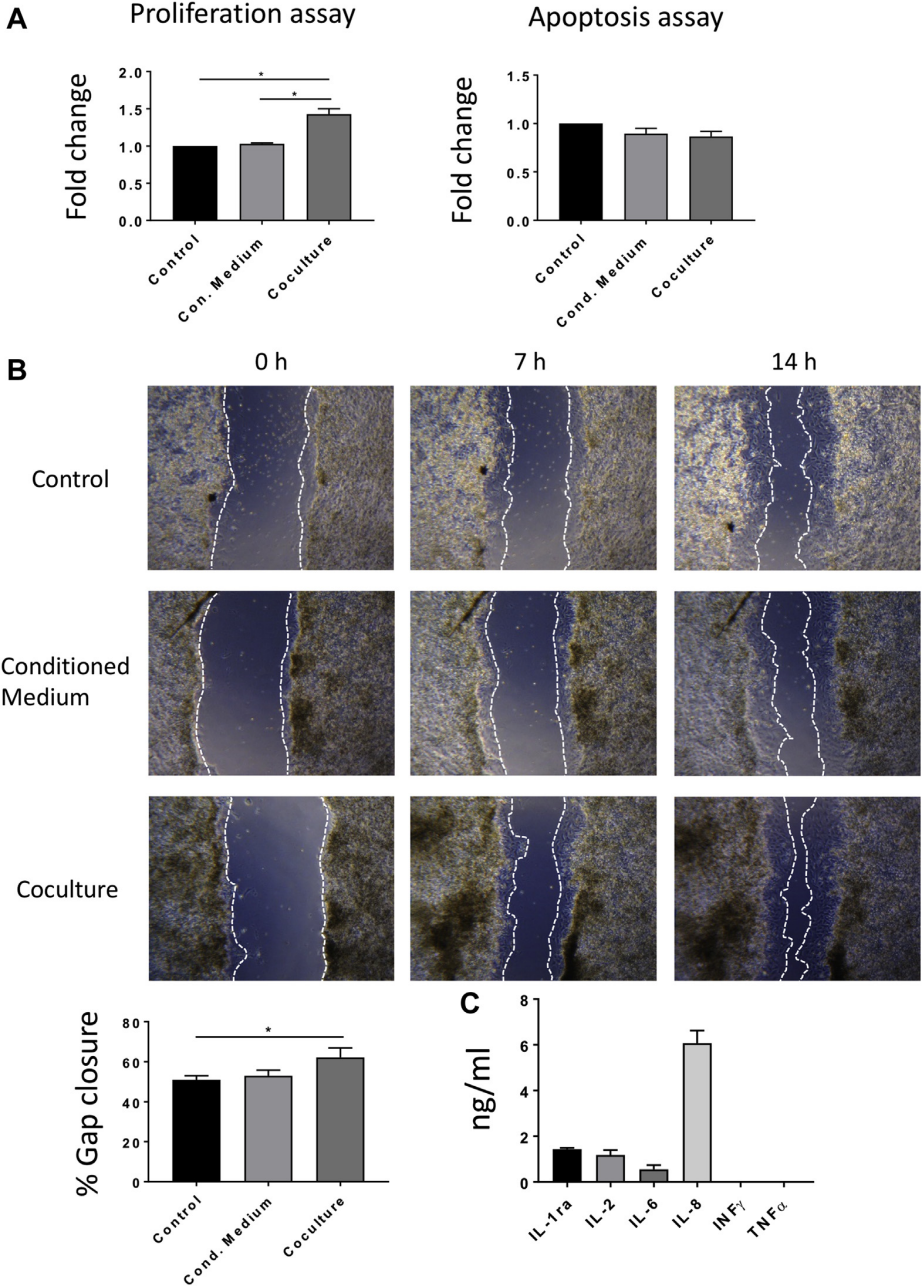
in Vitro Assessment of the Paracrine Effect of the Thymus-Derived Mesenchymal Stem Cells **(A)** Evaluation of the proliferation and apoptosis of rat cardiomyocytes (rCMs) cultured in the presence of thymus-derived mesenchymal stem cells (T-MSCs) or with their conditioned medium showed that the presence of the stem cells stimulates the proliferation of the cardiomyocytes (CMs), without affecting apoptosis (n = 3). **(B)** A migration assay of the rCMs either cultured with the conditioned medium or cocultured with the T-MSCs demonstrated that the stem cells can promote the migration of the CMs. The proportion of gap closure 14 h following the scratch was greater in the cocultured condition compared with the control. **(C)** Analysis of the cytokines and growth factors released by the T-MSCs showed secretion of interleukin (IL)–1ra, IL-2, and IL-6 and a high level of IL-8. Absence of interferon (INF)-γ and tumor necrosis factor (TNF)–α was observed in the porcine T-MSC (pT-MSC)–conditioned medium.

To assess the paracrine properties of T-MSCs, we measured the proliferation and apoptosis of CMs in the presence of T-MSCs or their conditioned medium (Figure 8A). Results showed stimulation of CM proliferation by T-MSCs in a coculture system (p = 0.0489 vs. control), whereas the T-MSC-conditioned medium was ineffective (p = 0.9901 vs. control). T-MSCs or their conditioned medium did not affect CM apoptosis (control vs, conditioned medium; p = 0.2588; control vs. coculture; p = 0.1244). Therefore, continuous production of paracrine factors by T-MSCs is indispensable for induction of CM expansion in vitro. Similarly, a migration assay of the CMs either cultured with the T-MSC-conditioned medium or cocultured with the T-MSCs confirmed that the stem cells have the potential to trigger the migration of CMs in vitro (Figure 8B). Fourteen hours after the scratch was made, the proportion of gap closure in the coculture system was higher compared with the control subject (62.18 ± 4.69% and 51.06 ± 2.01%, respectively; p = 0.0487 vs. control subject).

A multiplex analysis of the cytokines produced by the T-MSCs showed that high levels of the proangiogenic factor IL-8 are secreted by the MSCs in vitro (Figure 8C). Relatively low levels of inflammatory IL-2 and IL-6 and the anti-inflammatory cytokine IL-1ra were detected, whereas complete absence of proinflammatory interferon-γ and tumor necrosis factor–α was revealed.

## Discussion

Because of their similarities to humans and rapid growing rate, pigs are valuable animal models for preclinical assessment of cardiac tissue engineering 12, 13. The present study confirms the feasibility and safety of RVOT reconstruction using a SIS-ECM graft in growing piglets. In addition, we report for the first time that the addition of allogeneic T-MSCs to the SIS-ECM can upgrade the scaffold, converting it into a living tissue, capable of remodeling and improving RVOT contractility. Immunostaining of the explants showed similar neovascularization but a lower degree of fibrosis and a greater abundance of mature myocytes in the group implanted with seeded grafts. Moreover, scanning electron microscopy and immunohistochemistry analysis demonstrated a more organized endothelialization of the T-MSC-engineered grafts.

Very few studies have focused on reconstruction of the RVOT in animal models. Tanaka et al. (14) implanted a SIS-ECM graft allowing the controlled release of basic fibroblast growth factor in an RVOT porcine model. They reported the occurrence of regional myocardial remodeling and increased tissue viability within the patch. However, there was no improvement in the RVOT strain. Thus, our SIS-ECM cellularization approach seems to have a significant functional advantage over basic fibroblast growth factor treatment of SIS-ECM. In another study, induced pluripotent stem cells were used to engineer a synthetic scaffold that was implanted in the RVOT of a rat model (3). Host CM regeneration was observed in the cell-seeded group.

SIS-ECM material has been widely used in cardiovascular surgery since 2006. Early results suggested a therapeutic potential for correction of congenital heart defects 1, 15. However, more recent data have demonstrated less favorable clinical outcomes. For instance, Woo et al. (16) showed a relatively high degree of chronic inflammation and fibrosis and poor graft recellularization and integration with the surrounding tissue in SIS-ECM specimens explanted from pediatric patients who underwent corrective heart surgery. Similar concerns were raised by studies using SIS-ECM for valve repair in children. An intense inflammatory response was observed in the surrounding tissue, and little or no remodeling was detected 9 months after implantation (17).

Our group recently showed that T-MSCs isolated from human donors are capable to engraft and proliferate onto the SIS-ECM (2). We have now established the isolation and expansion of porcine T-MSC lines suitable for use in an allogeneic or autologous setting. The porcine T-MSCs displayed plastic adherence, a high rate of growth, high expression of the typical mesenchymal markers, and trilineage differentiation capacity into osteocytes, adipocytes, and chondrocytes (18). Hence, our results indicate that the isolation of MSCs from the porcine thymus is feasible and provides a homogenous cell population. Successful seeding of the T-MSCs on the SIS-ECM allowed the generation of an engineered patch composed of a multilayer of oriented cells growing on the surface of the scaffold. Mechanical testing of the construct showed that T-MSC incorporation is not detrimental to the graft physical properties. Furthermore, Y chromosome fluorescent in situ hybridization staining on seeded grafts explanted 24 h, 1 week, and 2 weeks post-operatively indicated that the donor cells engrafted into the SIS-ECM. However, Y chromosome staining on the 4.5-month explant demonstrated no trace of the implanted cells. This implies that the effects exerted by the cells on the surrounding tissue are due mainly to a paracrine stimulation that must have happened relatively soon after implantation. In line with our results, Sugiura et al. (3) showed that the seeded cells disappeared from the graft 4 weeks after implantation. Nonetheless, their early presence in vivo was sufficient to favor the regeneration of cardiac tissue.

The analysis of graft remodeling provided a possible interpretation for the improvement of RVOT contractility. The reduction of fibrosis associated with an increase in CMs’ number may have contributed to rescue RVOT strain in animals implanted with T-MSC-seeded grafts. We excluded the possibility that the CMs colonizing the graft derived from the donor cells, as none were found to be positive for the Y chromosome at the latest time point. Migration or proliferation of CMs from the neighboring myocardium could have contributed to the muscularization of the graft. In vitro studies showed that T-MSCs have the potential to stimulate the proliferation and migration of neonatal CMs. However, in agreement with the notion that adult CMs are terminally differentiated, we did not find any sign of CM proliferation in the explanted grafts, the only Ki67-positive cells being vascular and stromal cells. Therefore, CM migration remains the most likely explanation for graft muscularization in vivo. Furthermore, only seeded grafts induced the colonization of CMs coexpressing cMYH and Cx-43, while unseeded grafts were unable to maintain normal RVOT strain and contained cMYH cells negative for Cx-43. This marker has been associated with preserved CM functionalities. For instance, early-stage compensated left ventricular hypertrophy is characterized by increased Cx-43 immunofluorescent signal per myocyte volume and extensive Cx-43 lateral labeling, whereas Cx-43 becomes down-regulated in the decompensated stage of cardiac hypertrophy (19). Gap junctional coupling is important for functional integration of transplanted cells with host myocardium (20). Furthermore, noncanonical functions of Cx-43 include the control of sodium channels with important repercussions on the propagation of electric activity in the heart (21).

Graft cellularization resulted in a more organized endothelialization of the endocardial side of the graft but did not improve revascularization at capillary or arteriole level. Interestingly, arteriogenesis was not associated with a robust capillarization of the graft. Hence, it would be important to determine in the future whether stimulation of angiogenesis could further improve RVOT functionalization.

## Conclusions

Our study indicates that functionalization of the graft with T-MSCs may obviate the contractility and endothelialization problems reported after surgical correction of the RVOT (10). The addition of T-MSCs to the SIS-ECM converted it into a living tissue, capable of preventing the formation of an akinetic RVOT area. Akinetic regions developed in the RVOT have been associated with myocardial scarring, fibrosis, and electromechanical delay (22). Furthermore, abnormal fibrous tissue deposition and adipose tissue substitution can develop around the surgical scar and can eventually lead to electric instability, re-entrant arrhythmias, and even sudden cardiac death 23, 24. We did not perform electrophysiological studies; thus, we can only infer the absence of life-threatening arrhythmias from the fact that no fatality was observed. Another limitation of the study was the absence of a sham-operated group to assess unspecific background effect of surgery on the heart. However, the main comparator was the group of pigs with grafts that were not seeded with stem cells.

Most of the grafts currently used in cardiac surgery are either synthetic or fixed materials. These materials have not been proved to be particularly effective during long-term follow-up, leading to lack of contractility and mismatched mechanical properties, which might contribute to the risk for sudden death 25, 26. Our feasibility, safety, and efficacy in vivo study has shown that seeding SIS-ECM with T-MSCs overcomes some of the known limitations of current prosthetic material. These results may pave the way to new modalities for effective surgical restoration of RV function in patients with tetralogy of Fallot.

Perspectives **COMPETENCY IN MEDICAL KNOWLEDGE:** Our study demonstrates for the first time in a clinically relevant, randomized, large animal study the importance of using autologous stem cell tissue engineered patches for reconstruction of the RVOT.

**TRANSLATIONAL OUTLOOK:** The translation of this work into a clinical safety and efficacy study in patients with tetralogy of Fallot can open the way to the application of personalized grafts for reconstructive surgery in congenital heart disease, which can grow and remodel in vivo and therefore avoid the life-threatening complications seen in these complex patients. Our main challenges will be to transfer the production of our tissue-engineered grafts into clinically upgraded facilities and to identify funding for this very new and exciting “personalized” approach for neonates and infants born with congenital heart disease.
