# Supplementary material for: Successful Reconstruction of the Right Ventricular Outflow Tract by Implantation of Thymus Stem Cell Engineered Graft in Growing Swine
Source: JACC Basic Transl Sci. 2019 Jun 24;4(3):364–84. doi: 10.1016/j.jacbts.2019.02.001 (PMC6609916; doi:10.1016/j.jacbts.2019.02.001)

ONLINE APPENDIX

Online Supplementary Figure 1. Schematic diagram showing all the steps and techniques performed in the study.

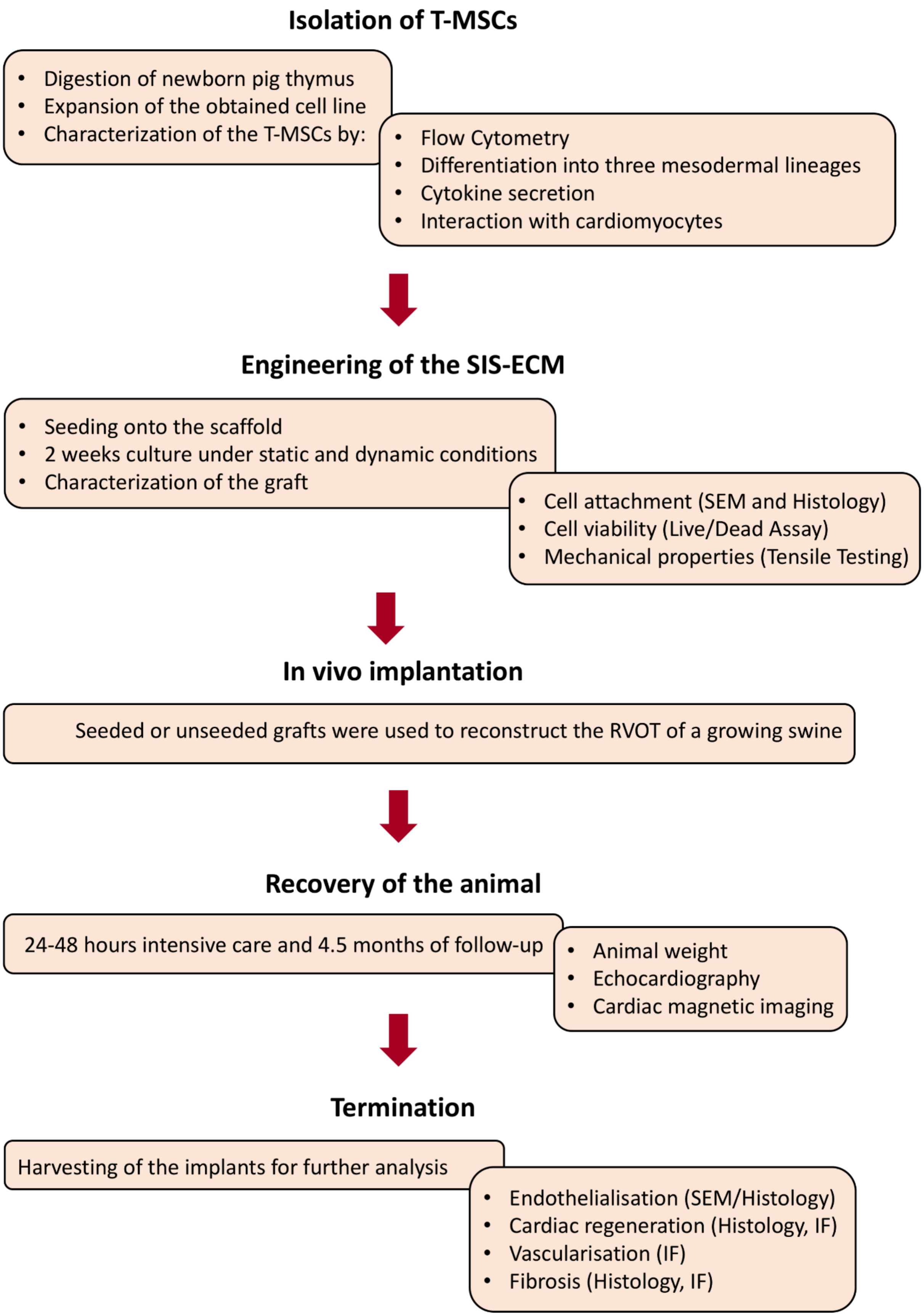
Isolation of T-MSCs
• Digestion of newborn pig thymus
• Expansion of the obtained cell line
• Characterization of the T-MSCs by:
• Flow Cytometry
• Differentiation into three mesodermal lineages
• Cytokine secretion
• Interaction with cardiomyocytes
Engineering of the SIS-ECM
• Seeding onto the scaffold
• 2 weeks culture under static and dynamic conditions
• Characterization of the graft
• Cell attachment (SEM and Histology)
• Cell viability (Live/Dead Assay)
• Mechanical properties (Tensile Testing)
In vivo implantation
Seeded or unseeded grafts were used to reconstruct the RVOT of a growing swine
Recovery of the animal
24-48 hours intensive care and 4.5 months of follow-up
• Animal weight
• Echocardiography
• Cardiac magnetic imaging
Termination
Harvesting of the implants for further analysis
• Endothelialisation (SEM/Histology)
• Cardiac regeneration (Histology, IF)
• Vascularisation (IF)
• Fibrosis (Histology, IF)

Online Supplementary Figure 2. Histological stains of the unseeded and seeded grafts. (a) Elastic van Gieson stain shows a collagen-rich tissue that constitute the graft side of the explants. The endocardial side also developed an endothelium containing a layer of elastin. (b) Von Kossa stain demonstrates lack of calcification in both the unseeded and seeded samples.

a

Elastin van Gieson

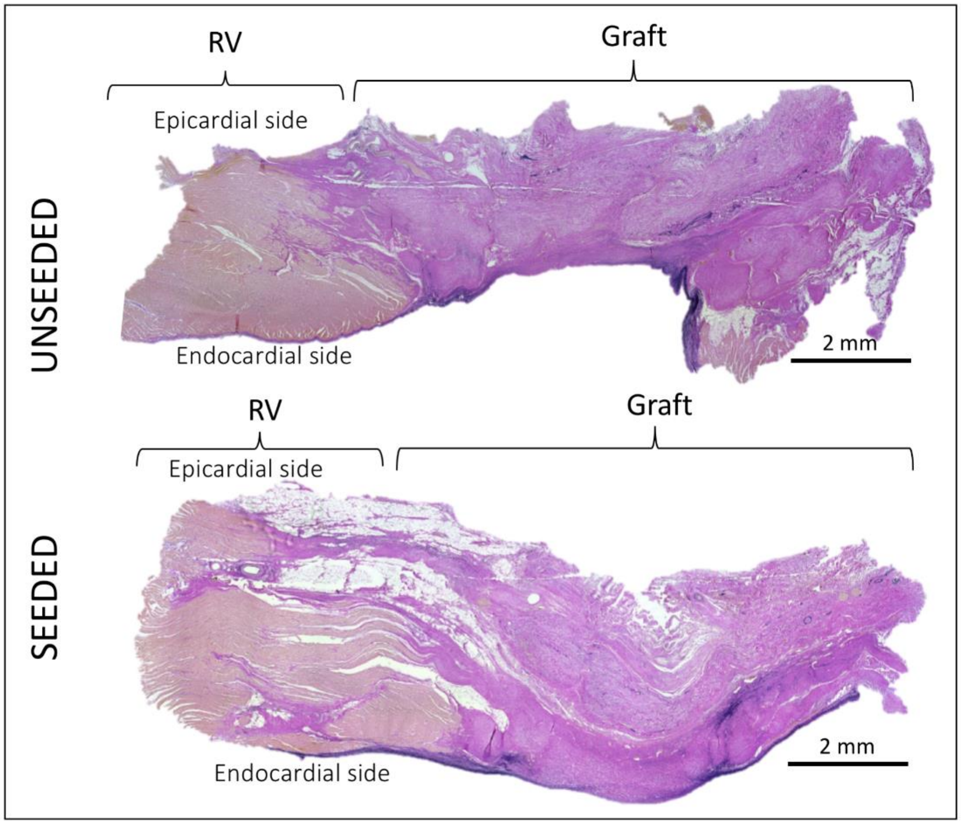

b

Von Kossa

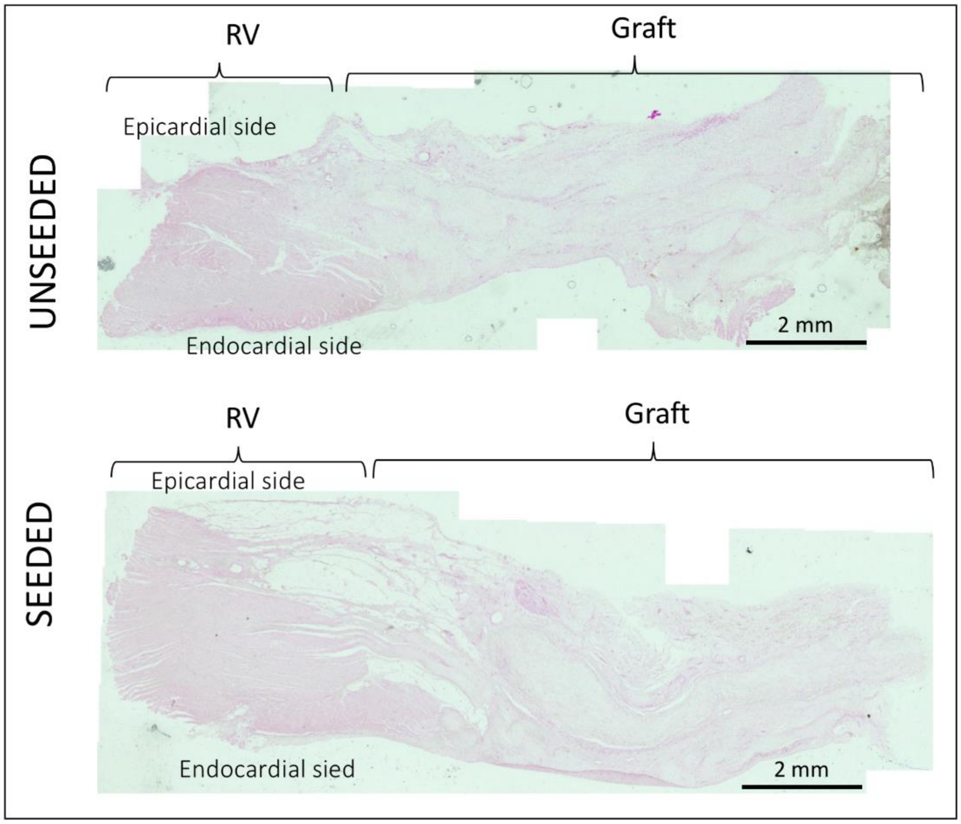

Supplement: Supplemental Figures 1 and 2 [file mmc1.pdf]
